# The effect of incidental dose to pelvic nodes in bladder-only irradiation in the era of IMRT: a dosimetric study

**DOI:** 10.1007/s00066-024-02246-2

**Published:** 2024-06-18

**Authors:** Gokhan Ozyigit, Alper Kahvecioglu, Mustafa Cengiz, Fazli Yagiz Yedekci, Pervin Hurmuz

**Affiliations:** 1 Faculty of Medicine, Department of Radiation Oncology, Hacettepe University, Ankara, Turkey; 2 Oncology Institute, Department of Radiation Oncology, Hacettepe University, 06100 Sıhhiye–Ankara, Turkey

**Keywords:** Bladder preserving therapy, Elective nodal irradiation, Trimodality treatment, Radiotherapy, Chemoradiotherapy

## Abstract

**Purpose:**

While three-dimensional radiotherapy (RT) causes high incidental nodal doses in bladder-only irradiation for muscle-invasive bladder cancer (MIBC), the impact on pelvic lymphatics is unclear in the era of intensity-modulated RT (IMRT). This study evaluates incidental doses to pelvic lymphatics in MIBC patients treated with IMRT.

**Methods:**

The data of 40 MIBC patients treated with bladder-only IMRT and concurrent chemotherapy were retrospectively evaluated. The pelvic lymphatics were contoured on initial simulation images and incidental nodal doses were evaluated. The Statistical Package for the Social Sciences (SPSS) version 23.0 (IBM, Armonk, NY, USA) was used for statistics.

**Results:**

Median RT dose to the bladder was 60 Gy in 30 fractions. In dosimetric analysis, median values of mean dose (D_mean_) of the obturator, presacral, external iliac, internal iliac, and distal common iliac lymphatics were 33 Gy (range 4–50 Gy), 3 Gy (range 1–28 Gy), 9.5 Gy (range 3–41 Gy), 7.5 Gy (range 2–14 Gy), and 1 Gy (range 0–15 Gy), respectively. The D_mean_ of the obturator lymphatics was significantly higher (*p* < 0.001) and the D_mean_ of the distal common iliac lymphatics was significantly lower (*p* < 0.001) than all remaining lymphatic stations. The D_mean_ of the external iliac lymphatics was significantly higher than that of the presacral lymphatics (*p* < 0.001), but the difference with the internal iliac lymphatics was not statistically significant (*p* = 0.563).

**Conclusion:**

The incidental nodal doses with bladder-only IMRT are heterogeneous and remain below the generally accepted doses for microscopic disease eradication for bladder cancer.

## Introduction

Although neoadjuvant platinum-based combined chemotherapy (CHT) followed by radical cystectomy (RC) is accepted as the gold standard for non-metastatic muscle-invasive bladder cancer (MIBC), organ-preserving approaches, such as trimodality therapy (TMT) including maximal transurethral resection (TUR) followed by definitive chemoradiotherapy (CRT), are another tempting alternative [1–3]. There is no prospective randomized data comparing TMT with RC. However, results of several studies indicate that TMT provides similar survival rates as compared with modern RC series in selected patients [4–8].

Although there is no clear consensus regarding the elective nodal irradiation (ENI) of pelvic lymphatics for patients without lymphatic metastasis, most of the Radiation Therapy Oncology Group (RTOG) trial protocols include ENI as a part of TMT [3]. On the other hand, there are also centers that do not routinely apply ENI in their protocols due to the lack of randomized data regarding the benefit of ENI [9–11]. However, even in the absence of ENI, pelvic lymphatics may be exposed to incidental radiation doses that may have the effect of eradicating microscopic disease, due to the proximity of target volumes. In a retrospective study which evaluated 20 patients treated with TMT but without ENI, the incidental mean dose (D_mean_) of the obturator, external iliac, and internal iliac lymphatics was found to be as high as 59 Gy, 45 Gy, and 36 Gy, respectively [12]. However, the radiotherapy (RT) technique employed in that study was three-dimensional conformal radiotherapy (3DCRT), and it is likely the primary cause of high incidental nodal doses.

Currently, it has become possible to apply highly conformal treatments with modern RT techniques such as intensity-modulated RT (IMRT) and volumetric modulated arc therapy (VMAT). Therefore, it is unclear whether the high incidental nodal doses obtained by 3DCRT are also valid in the era of IMRT and VMAT. This study aimed to evaluate incidental radiation doses to the pelvic lymphatics in MIBC patients received IMRT or VMAT without ENI.

## Materials and methods

### Patient population

The patients with non-metastatic MIBC treated with TMT in our department between January 2017 and December 2022 were included in this study. All patients had an initial thoracic and abdominal computed tomography ± positron-emission tomography/computed tomography (PET/CT) for staging, underwent a maximal TUR before CRT, and were treated with dynamic IMRT or VMAT techniques of RT. Patients receiving 3DCRT were excluded from the study. None of the patients had clinically suspicious pelvic lymph nodes before TMT. This study was conducted in compliance with the principles of the Helsinki declaration and approved by the institutional review board (Approval number: SBA 24/113).

### Radiotherapy details

The computed tomography simulation (CT-sim) was performed with a 2.5-mm slice thickness in two phases, one with a full bladder and the other with an empty bladder. For standardization of bladder filling, all patients were instructed to drink 500 cc of water 20–30 min before CT-sim. In our institutional policy, we routinely perform a two-phase irradiation for patients with MIBC [13]. In the first phase, we delineate the full whole bladder ± prostate as the clinical target volume (CTV)-1. The planning target volume (PTV)-1 is created as CTV-1 + 0.5–1.5 cm. The first prescription dose is 46–50.4 Gy in 23–28 fractions to PTV‑1. For the second phase of irradiation, CTV‑2 is delineated as the empty whole bladder, and PTV‑2 is delineated as CTV-2 + 0.5–1 cm. The second prescription dose is 9–14 Gy in 5–7 fractions.

We prescribed a total dose of at least 60 Gy for all patients. Before treatment, the same bladder filling protocol was performed. Daily cone-beam computed tomography was also performed for all patients during the CRT. Patients received concurrent weekly gemcitabine (50 mg/m^2^) during the treatment.

### Delineation of lymphatic stations

**Fig. 1:**
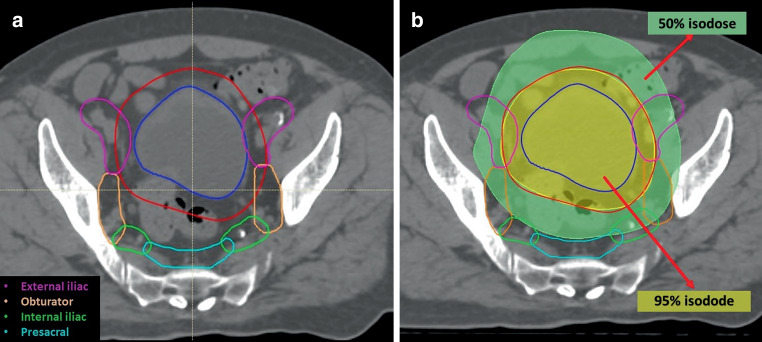
Delineation of pelvic lymphatics (**a**) and dose distribution of radiotherapy (**b**)

The delineation of pelvic lymphatic stations was retrospectively conducted based on CT-sim images of patients initially treated with bladder-only irradiation, following international consensus guidelines [14]. The obturator, internal iliac, external iliac, presacral, and distal common iliac lymphatics were delineated separately on CT-sim images (Fig. 1a). Subsequently, planning parameters such as the mean D_mean_, minimum (D_min_), and maximum (D_max_) doses of the lymphatic stations were evaluated (Fig. 1b).

### Follow-up

All patients underwent a cystoscopic evaluation at the sixth week after IMRT. Patients with complete response were monitored every 3 months during the first 2 years, every 6 months for the next 3 years, and annually thereafter.

### Statistical analysis

The Statistical Package for the Social Sciences (SPSS) version 23.0 (IBM, Armonk, NY, USA) was used for all statistical analysis. The follow-up period was defined from the starting day of CRT to the last follow-up, death, or recurrence, whichever came first. Locoregional control (LRC) was defined as the absence of either a locally recurrent tumor in the bladder confirmed by cystoscopy or pelvic lymph node metastasis detected radiologically. Kruskal–Wallis test was used to compare incidental doses of different pelvic lymphatic stations. The Mann–Whitney U test was also performed to test the pairwise differences using Bonferroni correction. The Kaplan–Meier method was used for survival analysis. A *p*-value < 0.05 was considered statistically significant.

## Results

### Patient, tumor, and treatment characteristics

Baseline patient, tumor, and treatment characteristics are summarized in Table 1. The median age was 69 years (range 43–88 years). Tumor histology was invasive urothelial carcinoma in all patients. Thirty-six patients (90%) had a pT2N0M0 tumor and four patients (10%) had a pT3N0M0 tumor according to the American Joint Committee on Cancer (AJCC) 8th Edition. The median total IMRT dose was 60 Gy (range 59.4–62 Gy). All patients received concurrent weekly gemcitabine at a median 6 cycles (range 3–6 cycles) with a weekly dose of 50 mg/m^2^. None of the patients received neoadjuvant or adjuvant CHT and/or immunotherapy.

**Table 1:** Patient, tumor, and treatment characteristics

Characteristic	Number (%)
*Age (median)*	69 years (range 43–88 years)
*Gender*	
Male	31 (78)
Female	9 (22)
*Stage (AJCC 8th edition)*	
II (pT2N0M0)	36 (90)
IIIA (pT3N0M0)	4 (10)
*Total dose of RT (median)*	60 Gy (range 59.4–62 Gy)
*Number of fractions (median)*	30 (range 30–33)
*PTV‑1 margin (median)*	1 cm (range 0.5–1.5 cm)
*PTV‑2 margin (median)*	1 cm (range 0.5–1 cm)

*AJCC* American Joint Committee on Cancer, *PTV* planning target volume, *RT* radiotherapy

### Recurrence patterns and survival

Median follow-up was 24 months (range 6–62 months). Two (5%) patients experienced a local recurrence (LR), a regional recurrence (RR) in the obturator lymphatic station was detected in one patient (2.5%), and four patients (10%) experienced distant metastasis (DM). The 1‑ and 2‑year LRC rates were 97 and 92%, respectively. The 2‑year overall survival (OS) and disease-free survival (DFS) rates were 97 and 87%, respectively.

### Dosimetric evaluation and comparison of pelvic lymphatics

The incidental radiation doses of the pelvic lymphatics are summarized in Table 2. The median D_mean_ values of the obturator, presacral, external iliac, internal iliac, and distal common iliac lymphatics were 33 Gy (range 25–50 Gy), 3 Gy (range 1–28 Gy), 9.5 Gy (range 3–41 Gy), 7.5 Gy (range 2–14 Gy), and 1 Gy (range 0–15 Gy), respectively. The median volume receiving a dose of at least 40 Gy (V_40 Gy_) of the obturator, presacral, external iliac, internal iliac, and distal common iliac lymphatics was 47% (range 13–88%), 3% (range 0–45%), 13% (range 5–63%), 9% (range 0–43%), and 1% (range 0–31%), respectively.

**Table 2:** Incidental doses of pelvic lymphatic stations

	Median D_mean_ (Gy)	Median D_min_ (Gy)	Median D_max_ (Gy)	Median V_40 Gy_ (%)
Obturator	33 (range 4–50)	7 (range 2–37)	54 (range 54–68)	47 (range 13–88)
Presacral	3 (range 1–28)	1 (range 0–17)	7 (range 2–38)	3 (range 0–45)
External iliac	9.5 (range 3–41)	1 (range 0–19)	34 (range 14–60)	13 (range 5–63)
Internal iliac	7.5 (range 2–14)	1 (range 0–15)	31 (range 20–56)	9 (range 0–43)
Distal common iliac	1 (range 0–15)	<1 (range 0–3)	<1 (range 0–37)	1 (range 0–31)

*D*_*max*_ maximum dose, *D*_*mean*_ mean dose, *D*_*min*_ minimum dose, *V*_*40*_ _*Gy*_ volume exposed to a minimum of 40 Gy

The D_mean_ of the obturator lymphatics was significantly higher (*p* < 0.001) and the D_mean_ of the distal common iliac lymphatics was significantly lower (*p* < 0.001) than those of all remaining lymphatic stations. The D_mean_ of the external iliac lymphatics was significantly higher than that of the presacral lymphatics (*p* < 0.001), but the difference to the internal iliac lymphatics was not statistically significant (*p* = 0.563). The D_mean_ values of the internal iliac and presacral lymphatics were also not statistically significantly different from each other (*p* = 0.711).

**Fig. 2:**
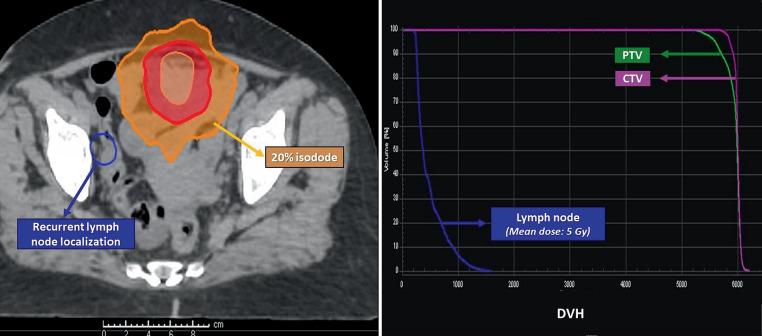
Dose distribution and dose–volume histogram of the patient with regional failure. *CTV* clinical target volume, *PTV* planning target volume

For patient with a regional failure, the computed tomography showing the recurrent lymph node was registered with the initial CT-sim, and the D_mean_ of this recurrent lymph node region was observed to be 5 Gy (Fig. 2).

## Discussion

In this study, we show that the incidental pelvic nodal doses are very low with IMRT and VMAT techniques in patients with nonmetastatic MIBC receiving TMT with bladder-only irradiation. However, it is noteworthy that the rate of regional failure in our cohort is quite low despite inadequate incidental nodal doses for microscopic disease eradication.

Trimodality therapy, as an organ-preserving approach, is currently one of the most commonly applied treatments in eligible patients and includes maximal TUR followed by definitive CRT [15]. Although there is currently no prospective randomized study comparing radical cystectomy with TMT, it has been stated that both treatment modalities provide similar survival rates based on the results of several studies [3, 4, 11, 16]. Although TMT is currently recommended by many centers for patients who met the eligibility criteria for an organ-sparing approach, TMT protocols also vary widely between centers. The benefits of concurrent CHT for medically fit patients and maximal TUR before CRT are well defined for patients receiving TMT and do not vary much worldwide [11]. On the contrary, there is no optimal standard protocol for simulation, target volume delineation, and dose prescription. Previously, we have reported our gemcitabine-based TMT outcomes without ENI for patients with non-metastatic MIBC [13]. However, the RT techniques employed in that study were either 3DCRT or IMRT/VMAT.

In a dosimetric study of 20 patients, pelvic lymphatics received high radiation doses incidentally in patients undergoing 3DCRT, even without ENI [12]. Approximately 35% of the failure patterns in patients treated with TMT are locoregional recurrences ± distant metastasis [17]. Among the underlying reasons why the survival benefit of ENI has not yet been proven in any studies and its role in TMT is controversial, there is the possibility of incidental irradiation of pelvic lymphatics in patients receiving bladder-only irradiation [18, 19]. In the results of a recently published large National Cancer Database study from the United States, ENI did not improved OS as compared with bladder-only irradiation for 2104 patients with T2-T4N0M0 MIBC. However, it should be taken into consideration when evaluating these results that 83.7% of the patients who received bladder-only irradiation were treated with a 3DCRT technique, and incidental nodal doses may be sufficient for microscopic disease eradication, at least in a significant portion of patients. On the other hand, IMRT and VMAT provide better OAR preservation as compared to 3DCRT, and incidental nodal doses may not be as high as in 3DCRT with IMRT and VMAT techniques.

The largest bladder-sparing trial to date, the Bladder Cancer 2001 (BC2001) trial from the United Kingdom, demonstrated the benefit of addition of CHT to RT for both locoregional control and disease-free survival [10]. Unlike most of the RTOG studies, ENI was not performed in the BC2001 trial and bladder-only irradiation was administered to the patients. The regional failure rate was found to be approximately 5% [20, 21]. However, the RT technique used in the BC2001 trial was also 3DCRT. Thus, the possibility of incidental nodal irradiation should be taken into consideration when interpreting the results. Currently, no data exist comparing IMRT- or VMAT-based bladder-only irradiation vs. bladder + ENI approaches. To the best of our knowledge, our study is the first to investigate incidental nodal doses with bladder-only irradiation in the era of IMRT and VMAT. The results of our study show that incidental nodal doses with bladder-only irradiation applied with modern techniques are lower than in historical 3DCRT series. Despite low incidental nodal doses, the regional failure rate was only 2.5% in our study. On the other hand, the only lymphatic recurrence developed from a low-dose-painted area. In the study by Baumann et al. [22], the 5‑year pelvic recurrence rate was reported as 28% in patients with ≥ pT3 tumors and as 8% in patients with ≤ pT2 tumors after radical cystectomy. They proposed that in cases of stage ≥ pT3 with negative margins, RT should include targeting the iliac and obturator nodes at a minimum. Additionally, for cases of stage ≥ pT3 with positive margins, coverage of the presacral nodes and cystectomy bed may be required. On the other hand, since patients treated with TMT generally have T2 tumors, it remains uncertain from the current literature whether there is a particular lymph node level that requires a higher radiation dose. Although there is no universally accepted standard definition of radiation dose for microscopic disease eradication, there are studies demonstrating the effectiveness of doses ranging from 30 to 50 Gy in various tumor types [23, 24]. Hence, in our study, we also analyzed the volumes of V40 Gy, revealing that even in the obturator lymphatic region, where this ratio peaks, it remained below 50% of the prescribed dose with IMRT and VMAT.

In addition to ENI, there are also surgical studies examining the role of treatment intensification towards lymphatics on oncological outcomes. The role of extended pelvic lymphadenectomy (EPL) during radical cystectomy was evaluated in a recent phase III randomized controlled trial, SWOG-1011, the results of which were presented at the American Society of Clinical Oncology 2023 Annual Meeting (NCT01224665). In standard lymphadenectomy, the obturator, external iliac, and internal iliac lymphatics were removed. In EPL, the common iliac, presciatic, and presacral lymphatics were also removed in addition to the standard lymphadenectomy. In the results, EPL did not improve OS and DFS as compared with standard lymphadenectomy. In addition, EPL was also associated with higher rates of toxicity.

Although our study is the first to show that high incidental nodal doses are not valid in the era of IMRT/VMAT, and current margin concepts for patients receiving TMT with bladder-only irradiation, it also has some limitations. First, the small retrospective cohort size with a short follow-up limits the generalizability of our findings. Secondly, various clinical and pathological features that may be related to regional recurrence could not be evaluated, and the low regional recurrence rate in our findings requires careful interpretation due to the focus on dosimetric analyzes of the study.

## Conclusion

Even with a full-bladder simulation, the incidental pelvic nodal doses with IMRT/VMAT in non-metastatic MIBC patients treated with bladder-only irradiation remain below the generally accepted doses for microscopic disease eradication. Although it is well known that 3DCRT causes high incidental nodal doses for bladder-only irradiation, these doses are quite low with more conformal RT techniques such as IMRT/VMAT, and should be considered when deciding whether to administer ENI or not. Consequently, prospective studies are required to evaluate the role of ENI in patients receiving only IMRT/VMAT, and to determine the optimal target volume delineation protocol for the identification of subgroups that may possibly benefit from ENI.
